# Barriers to HIV service utilisation by people living with HIV in two provinces of Zimbabwe: Results from 2016 baseline assessment

**DOI:** 10.4102/hivmed.v19i1.721

**Published:** 2018-08-09

**Authors:** Taurayi A. Tafuma, Nyikadzino Mahachi, Chengetai Dziwa, Tafara Moga, Paul Baloyi, Gladys Muyambo, Auxilia Muchedzi, Tinashe Chimbidzikai, Getrude Ncube, Joseph Murungu, Tendai Nyagura, Katherine Lew

**Affiliations:** 1 FHI 360, Zimbabwe; 2 Plan International, Zimbabwe; 3 Ministry of Health and Child Care, Zimbabwe; 4 United States Agency for International Development, Zimbabwe

## Abstract

**Background:**

The emergence of antiretroviral therapy (ART) transformed HIV from a terminal illness to a chronic disease. However, limited access to health services remains one of many barriers to HIV service utilisation by people living with HIV (PLHIV) in low-resource settings. The goal of this study was to describe the barriers to HIV service utilisation in two provinces of Zimbabwe.

**Methods:**

A qualitative descriptive study was conducted with PLHIV and village health workers (VHW) in eight districts within the two provinces. Convenience sampling was used to select the participants. This sampling was limited to communities supported by health facilities with more than 500 PLHIV enrolled into HIV care and treatment. Interviews were audio-recorded and transcripts were subjected to thematic content analysis.

**Results:**

A total of 22 community focus group discussions (FGDs) were conducted. Barriers to using HIV services cited in PLHIV and VHW FGDs were similar. These were categorised as health system-related barriers, which include user fees, long waiting times, lack of confidentiality and negative attitudes by healthcare providers, and lack of consistent community-based HIV services. Community-related barriers cited were stigma and discrimination, food insecurity, distance to facilities and counterproductive messaging from religious sectors. Client-related factors reported were inadequate male involvement in HIV-related activities and defaulting after symptoms improved.

**Conclusion:**

Our assessment has indicated that there are several barriers to the utilisation of HIV services by PLHIV in the two provinces of Zimbabwe. As new strategies and programmes are being introduced in the current resource-constrained era, efforts should be made to understand the needs of the clients. If programmes are designed with an effort to address some of these challenges, there is a possibility that countries will quickly achieve the 90-90-90 targets set by The Joint United Nations Programme on HIV/AIDS.

## Introduction

An effective community human immunodeficiency virus (HIV) response requires coordination and synergy among actors supporting HIV testing and treatment services, provision of compassionate care for people living with HIV (PLHIV), open and non-stigmatising discussions of HIV and concrete strategies to prevent new infections.^1^ However, with limited access to health services in low-resource settings, communities are not mounting a sufficient response. It has been noted that limited access to health services is recognised as one of the greatest barriers to entry into the healthcare system, hindering HIV testing, treatment and care.^2^ In areas where services are available PLHIV are faced with economic, geographic and social barriers to access those services. This array of setting-specific challenges resulted in the global burden of HIV being unevenly distributed, with sub-Saharan Africa having the greatest share.^3,4^ It has become imperative that efforts be made to have healthcare systems evolve and focus on providing patient-centred and high-value care. Research has shown that most patients prefer to be involved in medical decisions,^5^ which should include programming of their services. Involving clients in programming begins with developing an understanding of their needs and assessing barriers to the availed services.

Advances in antiretroviral therapy (ART) transformed HIV from a terminal illness to a chronic disease^6,7^ and resulted in significant decreases in HIV-related morbidity and mortality. In Zimbabwe, where there are approximately 1.4 million PLHIV, HIV services have been decentralised to increase accessibility for people.^8^ By June 2015 approximately 842 372 (60%) of PLHIV were on ART and 94% of health facilities were offering ART services.^8^ Despite the massive scale-up in national HIV testing programmes, studies suggest that up to 80% of HIV-infected adults do not know their status in some sub-Saharan African settings and only 47% of adults who are eligible are accessing ART, although rates vary substantially by country.^9^ There are many factors that can possibly explain these gaps, and currently most studies on the HIV cascade have been focused on clinic-level data.^10^

Community-level interventions are believed to create an enabling environment for the utilisation of HIV services.^10^ Zimbabwe was one of the first African countries to implement the holistic primary healthcare approach, which was adopted at the Alma Ata Conference of 1978 through the introduction of the village health worker (VHW) programme in 1981.^11^ A number of improvements have been made to this programme since then as a way of positioning communities to contribute meaningfully in the provision of healthcare, disease prevention and promotion of health and well-being.^11^ Zimbabwe, in its National Strategic HIV and AIDS Plan III (2015–2018),^12^ recognised that strengthening community participation ensures high standards of transparency, accountability of health service management and community ownership. However, to date, community system engagement and strengthening has been limited. Literature reports that most countries’ community response has not been sufficiently defined and prioritised, resulting in lack of cohesion and funding.^10^ Ultimately, this has contributed to the underutilisation of HIV services by PLHIV. In order to address some of these gaps in service utilisation, the US Agency for International Development (Zimbabwe) funded FHI 360 for the five-year Zimbabwe HIV Care and Treatment (ZHCT) project. The main goals of the project are (1) to increase the availability of quality comprehensive care and treatment services for PLHIV at community level and (2) to strengthen community-level health systems to monitor, track and maintain PLHIV in care. Understanding community-level factors in HIV care and treatment contributes to the larger effort in designing multilevel, effective and sustainable programmes and interventions for HIV epidemic control.^10^ We present here the qualitative findings from a baseline assessment that was designed to determine the barriers to HIV service utilisation by PLHIV in eight districts in two provinces of Zimbabwe.

## Methodology

This was a qualitative descriptive study design that used focus group discussions (FGDs) with PLHIV and VHWs. The study targeted eight districts in Manicaland and Midlands provinces in Zimbabwe, where FHI 360 was to implement the ZHCT project. These districts have high numbers of PLHIV (17 500–34 000) and the HIV prevalence among adults (15–49 years) is in the range of 9% – 20%. The provincial HIV prevalence among adults (15–49 years) in Manicaland and Midlands is 11% and 15.5%, respectively.^13^ Overall, Manicaland Province has the largest population of PLHIV^14^ in Zimbabwe. Convenience sampling was used to select participants for the FGDs and limited to communities supported by health facilities with more than 500 PLHIV enrolled into HIV care and treatment. PLHIV and VHWs were grouped into separate FGDs. This was to ensure that more FGDs were conducted in districts with more clients on ART. Data were collected from May to June 2016.

Village health workers (VHW) are the community-based cadres who coordinate activities related to health at village level and act as conduits into formal clinic-based care.^11^ PLHIV are individuals who are receiving HIV services (ART or pre-ART services) from the selected facilities. Participant recruitment was limited to clients 18 years and above and coordinated by field supervisors and representatives of facilities. Field supervisors approached selected facilities and informed facility representatives (nurse-in-charge) about the study. PLHIV were then informed of the study at the time of drug collection or review and those interested had their names documented as potential participants. VHWs who support facilities of interest were also informed about the study during their daily activities. The potential participants were then later contacted by field supervisors. At this stage, these participants were given detailed information describing the purpose of the research, confidentiality and the right to refuse to participate. Appointments were then set for the FGDs. Interviewers explored the existence of stigma and discrimination against PLHIV, existence of supporting services at community level and barriers and facilitators to accessing HIV services for PLHIV. As for the VHWs, we explored the perceived attitude of the community towards them, relations with health facilities and perceived barriers and facilitators in the uptake of HIV services by PLHIV.

Interviewers and field supervisors were trained on how to recruit and obtain consent from prospective participants and how to conduct FGDs. A total of eight interviewers were recruited, with four of them being males. All of them had experience in conducting FGDs and they had gone through the ethical training under FHI 360. In addition, the field supervisors were trained on organising, coordinating and supervising data collection activities in their respective sites, including data verification and how to remotely transfer data from their site to the provincial and central office. A pretest of the data collection tools was conducted in a district that was not under assessment. A total of 22 FGDs, each with 6–12 participants, were conducted (out of 24 planned). The distribution of FGDs was based on the number of facilities in a district with more than 500 PLHIV on ART per district. If more than 50% of the facilities in a district had more than 500 PLHIV, they were assigned four FGDs while those with below 50% were assigned two FGDs. The FGDs were conducted in quiet and discreet locations, often a vacant community hall or open space where community gatherings were usually held. These were places that were agreed on by the participants.

Data analysis was performed manually by three FHI 360 researchers, two from the Zimbabwe office and one from headquarters. Data from the FGDs were translated and transcribed by the interviewers from Shona and Ndebele to English. Transcripts were verified to ensure headings and content were correct. This included multiple readings of the transcripts developed to capture context and meaning, followed by coding and categorisation of recurring concepts and ideas. Data verification was done by a third researcher from headquarters, who also coded all transcripts. Codes were compared and added or removed based on the agreement between analysts. Once data were translated, transcribed and verified, audio recordings were deleted from the recording devices and hard- and soft-copy transcripts were stored securely and safely in lockable cabinets and on password-protected computers. Common themes were analysed using content analysis, grouped and quantified according to the volume of similar responses. No repeat interviews were carried out. Utilisation of HIV services in this case included uptake of HIV testing to staying on ART while participating in other community-related HIV activities such as advocacy, health promotion and education.

## Ethical consideration

Ethical approval was granted by the Medical Research Council of Zimbabwe (MRCZ) and FHI 360 Institutional Review Board (IRB). The study was conducted in compliance with protocol after permission was obtained from the Ministry of Health and Child Care (MoHCC). Further fieldwork clearance was sought from relevant local authorities and leadership at district level.

## Results

A total of 22 FGDs were conducted in eight districts, 10 with VHWs and 12 with PLHIV. A total of 106 PLHIV and 96 VHWs took part in the FGDs. Most (68%) of PLHIV who participated were women and the average age was 43 years (s.d. 10 years). Similarly, most of the VHWs were female (73%). The average age for VHWs was 43 years (s.d. 9 years). The perceived barriers reported by participants were categorised under the following three broad themes: barriers related to the health system; barriers related to clients; barriers in the broader community (these are issues experienced outside health facilities and are perpetuated by members in the community or experienced at community level where people live); and other barriers. The barriers described below were those reported most frequently.

### Health system-related barriers

**User fees at health facilities**: Participants in all districts felt that there was no good justification for payment of user fees at different levels of service provision. This was raised by the PLHIV FGD:

‘.… at clinic, one has to pay and pays again at referral hospital. What is the justification for these charges?’ (PLHIV FGD)

**Long waiting times at health facilities**: This was mentioned across all districts and was attributed to inadequate staffing, poor attitudes from healthcare providers and complicated referral processes within health facilities:

‘Nurses spend time socialising during working hours.’ (PLHIV FGD)

‘Some clients might give up accessing services after waiting so long before being served at health facilities.’ (VHW FGD)

**Lack of community-based HIV services**: This was mentioned in six districts, and PLHIV’s comments focused on VHWs being too busy or not active in some communities while VHW highlighted challenges with their voluntary work:

‘VHWs … should be monitored so that they provide their services. They also have multiple roles in the community, compromising the quality of their work.’ (PLHIV FGD)

‘They [*VHWs*] are not mobile and don’t come to us; if we want a service we go to their residence. We need them to visit us.’ (PLHIV FGD)

‘We were given bicycles and scales and they have long since stopped working … we are not paid incentives on time.’ (VHW FGD)

VHWs indicated that the nature of their work was mainly voluntary; hence they were less prioritised by authorities when payments and resupplies were being made. In one of the districts, respondents complained about the lack of incentives, airtime and transport, which made it difficult to perform their duties. This also contributed to the VHW programme having fewer males:

‘The lack of incentives is a barrier to attracting more men to be VHWs.’ (VHW FGD)

**Bad attitude by healthcare providers**: Nurses were singled out as having bad attitudes in all districts:

‘At clinics/hospitals that’s where all the stigma exists because nurses have attitudes; that’s why you will find some people will collect their pills from facilities away from their places.’ (PLHIV FGD)

However, not all were in agreement that nurses in some districts had bad attitudes:

‘There has been improvement in services and the way they treat us.’ (PLHIV FGD)

### Client-related barriers

**Inadequate male involvement in HIV-related activities**: Findings from both groups of FDGs were consistent on why men were less involved in HIV activities and were reported in all districts:

‘Men don’t participate … they are busy at work; they take time to accept their status; they only seek treatment when very sick; men just don’t like to be gathered around without financial benefit.’ (PLHIV FGD)

‘Some men are not accommodative; they feel male VHWs are lazy to work and thus opt for a female job.’ (VHW FGD)

**Defaulting after feeling better**: PLHIV reported that there were some individuals who would stop ART when they improved and this was reported in five districts:

‘People tend to relax after their health improves.’ (PLHIV FGD)

**Distance to health facilities**: The challenge of clients accessing care because of distance and related costs (transport) were mentioned in four districts, and this was also highlighted even if community HIV testing was provided:

‘If a person tests positive in the community and is far from health facilities, the linkage to care will be a challenge due to transport needs.’ (VHW FGD)

**Lack of trust**: It takes time for VHWs to establish themselves as a trusted cadre within the communities. Some community members were afraid that VHWs would disclose their health problems to the community. This resulted in VHWs being abused by some of the community members:

‘… we are verbally abused by those who do not want to disclose that you are the ones who are going to disclose our status to others.’ (VHW FGD)

Some mentioned that the behaviour of some of the VHWs was very bad, resulting in them not being trusted:

‘… their work is put into disrepute by some of their colleagues who display bad behaviour in the community, e.g. husband snatching, drunkenness, dishonesty.’ (PLHIV FGD)

### Community-related barriers

**Roles of religious leaders and traditional healers**: Counterproductive messaging was mentioned in six districts, with traditional or religious healers often convincing PLHIV that they do not need ART to manage HIV. Messages from religious leaders have been counterproductive to the efforts made by the Ministry of Health and Child Care (MoHCC) in managing HIV. The ‘white garment’ churches were singled out for this practice in two of the FGDs:

‘Churches are also causing followers to default, saying they have prayed for you and you have been healed.’ (PLHIV FGD)

‘Others go to prophets and replace their medication with anointed water from prophets.’ (PLHIV FGD)

‘We still have some members of white garment churches who are resistant; most members in those families died.’ (VHW FGD)

In some cases, people also opt for traditional medicine, rather than modern medicine, believing their HIV infection is a curse:

‘There are still people out there who believe that being HIV positive is a curse and would consult a traditional healer.’ (PLHIV FGD)

**Stigma and discrimination in the community**: Community-level stigma was listed as an important barrier to better treatment outcomes in five districts. PLHIV are still seen as unfaithful and are subjected to name-calling, particularly at social events:

‘People in the community pass negative comments once they found that one is HIV positive, and [*this*] is common at gatherings such as funerals and churches.’ (PLHIV FGD)

‘… we [*VHWs*] are verbally abused by PLHIV who feel that we disclose their HIV status to the community.’ (VHW FGD)

And, worryingly, respondents made a direct link between stigma and ART adherence:

‘Stigma still exists here, causing people not to go to collect their medications because they don’t want to be known.’ (PLHIV FGD)

**Food insecurity**: Food insecurity was mentioned in four districts, and this was mainly linked to the recent drought that Zimbabwe experienced. Lack of food was directly linked to ART adherence:

‘They also require food since they are supposed to take their drugs after they have eaten something.’ (PLHIV FGD)

### Other barriers

**Negative attitudes towards VHWs**: The VHWs mentioned that they were sometimes accused of stealing drugs, pressurising people too much to complete referrals and suspected of having intentions other than providing health education. This was raised in five districts:

‘People in the community give the impression that the health workers are out there to bother people and disturb them.’ (VHW FGD)

‘Our worst thing is that people talk bad about us if they find out that we don’t have the resources to assist them.’ (VHW FGD)

**Men as barriers**: There were many instances where men were highlighted as a significant barrier, especially within the families:

‘My husband gave me HIV and he did not want to get tested until he died. He was arrogant about getting tested. Men have a big problem and they want to keep on spreading HIV.’ (PLHIV FGD)

‘… we as females we are always complaining that our partners do not want to use protection when having sex even when they know they are positive.’ (PLHIV FGD)

‘The men do not want to visit clinics; this makes it difficult for women to disclose to their husbands … when they tell their husbands they are positive, at times they are blamed.’ (VHW FGD)

## The participants were also asked to make recommendations

Village health workers recommended that a system should be developed so that there is improved coordination between health facility staff and VHWs. Facilities should routinely share review dates with VHWs and they should give clients referred from VHWs first preference. Giving preference to referred clients might improve men’s participation in HIV services:

‘Men do not want to be kept waiting for long hours … and they do not want empty promises.’ (VHW FGD)

In addition, VHWs proposed availing food handouts to clients taking ARVs. This was also highlighted in the PLHIV FGDs:

‘… use the previous strategy where positives are enrolled and get food; this could promote men’s attendance.’ (PLHIV FGD)

‘People with HIV need food. We might lose many of them if help does not come.’ (VHW FGD)

Other recommendations were that X-rays for TB should be free for ART clients; user fees upon ART refills should also be removed; and health facilities, especially clinics, should have more nurses who are empathetic to clients. To improve male involvement in support groups, PLHIV suggested that these groups should carry out income-generating projects such as raising poultry. However, they suggested that these projects should be suggested by people themselves, rather than being dictated:

‘… mostly they would want to work for their families and hence can be lured into groups if there are opportunities for fundraising.’ (PLHIV FGD)

‘Income-generating projects should be decided by the people themselves and not dictated.’ (PLHIV FGD)

## Discussion

The above findings describe the perceived barriers to utilisation of HIV services in Manicaland and Midlands provinces. Findings from this assessment provide more information to programmes designed to provide HIV services at community level and strengthening facilitators currently existing with facility-based services.

Long waiting times at health facilities and the negative attitudes of healthcare providers highlighted above generally reflect the human resource constraints pervasive in busy ART clinics in sub-Saharan Africa.^15^ Long waiting times have been mentioned as a key driver in the attrition of clients on ART.^16^ Also of note is that competing activities such as work and social life among clients seeking medical attention tend to interfere with time spent queuing in overburdened health facilities. Introduction of flexible working hours is likely to improve uptake of services as has been noted in other studies.^17^ Although Zimbabwe has decentralised HIV services, user fees at some facilities are still a barrier to service utilisation, especially with the current economic challenges. PLHIV are likely to travel longer distances to access free services but this is not sustainable, as has been indicated in other settings.^2,18^ Community HIV services will address issues of long distances travelled by clients to access services and also decongest health facilities, resulting in shorter waiting times.

Our finding of a negative attitude among healthcare workers towards PLHIV is consistent with other studies and is associated with non-enrolment into care.^17^ Considering the sparsity of health services in rural areas of most of sub-Saharan Africa^2^ there is need to train health personnel in the importance of empathy towards patients, as well as engaging patients as partners in the HIV care process. This will enable PLHIV to visit their nearest facilities whenever they need HIV services and might indirectly improve adherence to treatment. Overall, this will help the country to achieve the second and third 90s of The Joint United Nations Programme on HIV/AIDS (UNAIDS) targets. Studies have shown that stigma is often measured at the individual level but it is a social construct that manifests itself at the community level.^10,17^ Generally, stigma has been associated with poor utilisation of HIV services^10^ and this was highlighted in five districts in this study. Fear of gossip and being called names results in PLHIV avoiding HIV services as well as disclosing. This has been reported to be prominent in several countries,^10,17^ and in Zimbabwe there are individuals who cannot confide to VHWs. Interestingly, Underwood et al.^10^ cited a study where stigma was a motivator of adherence as PLHIV took their medications to avoid a sickly appearance, which might draw community attention. However, stigma and discrimination should be discouraged regardless of this finding.

The fear of HIV disclosure among women, especially to their husbands, as highlighted, could be because of poor appreciation and utilisation of HIV services by males in these communities. A gender-balanced VHW programme would allow more males to be reached by their counterparts. Zimbabwe has embarked on couple testing and counselling also as a mitigating factor to this. However, its impact needs to be continuously assessed. With the introduction of household HIV index case testing in Zimbabwe, there is hope of strengthening disclosure of HIV status within households and improving utilisation of HIV services by all, as has been noticed in other studies.^17,19^ Overall, it is important to support strategies that address men’s challenges to access health services.

The reports from the FGDs that HIV spiritual healing is seen as a better alternative to medical management is not unique to these two provinces in Zimbabwe. Studies have shown that, at community level, religious organisations are influential social networks that have the power to support or stigmatise PLHIV and endorse or reject medical treatment of HIV.^20,21^ A study in Uganda noted that a few (1.2%) individuals discontinued their ART because they believed that their pastors’ prayers had cured them of HIV.^20^ Our findings were not quantified, hence we recommend quantitative studies to determine the impact of these counterproductive messages from religious sects. Manzou et al.^21^ highlighted the need for HIV programmes to strengthen collaboration with religious institutions so that appropriate messages are shared with PLHIV. Laws should be enacted to apprehend religious leaders who propagate spiritual healing of HIV.

Our participants made some important recommendations that could help address some of the gaps. There is need to increase collaboration between income-generating projects and HIV programmes so that men can be involved in HIV service provision. Food handouts for clients on ART was also suggested, although it had no effect on ART adherence in Mozambique.^22^ However, this could be different in Zimbabwe because of the current economic challenges. User fees at facilities should be removed for all HIV-related services. Most FGDs highlighted the need for personnel providing HIV care services to be trained in the importance of empathy towards PLHIV.

### Limitations

There are a number of limitations in our findings, including the fact that FGDs are known to elicit more views from verbal participants than shy ones. However, our teams were trained to moderate the discussions so as to mitigate this challenge. Recruitment of participants was done from sites with more than 500 PLHIV, and these were conveniently sampled from those already visiting health facilities, hence our results cannot be generalised. These clients might not have reported all barriers to accessing services, as they were receiving them at the time of recruitment. Furthermore, the age of the participants was high and we could have missed valuable information from the younger age groups. While we have made all attempts to maintain the integrity of our data, it is possible that some words or phrases were incorrectly translated, though we believe the substantive viewpoints were correctly inferred. One major strength of our assessment is that it highlights barriers perceived by the clients, who are the PLHIV, as well as their supportive cadres at community level.

## Conclusion

Our assessment has indicated that there are several barriers to the utilisation of HIV services by PLHIV in the two provinces of Zimbabwe. As new strategies and programmes are being introduced in the current resource-constrained era, efforts should be made to understand the needs of the clients. This will help in providing client-centred services. Although the barriers highlighted above could not be generalised, we have confidence that these are likely to be experienced across the country, as they were frequently cited in other settings. As such we recommend that MoHCC find ways to be responsive to the needs of PLHIV while improving the services of VHWs as conduits between communities and health facilities.
